# Activin A Secreted From Peripheral Nerve Fibroblasts Promotes Proliferation and Migration of Schwann Cells

**DOI:** 10.3389/fnmol.2022.859349

**Published:** 2022-07-07

**Authors:** Yan Li, Zhenghang Cheng, Fanhui Yu, Qi Zhang, Shu Yu, Fei Ding, Qianru He

**Affiliations:** ^1^School of Biology and Basic Medical Sciences, Soochow University, Suzhou, China; ^2^Key Laboratory of Neuroregeneration of Jiangsu and Ministry of Education, Co-innovation Center of Neuroregeneration, Nantong University, Nantong, China

**Keywords:** activin A, fibroblasts, Schwann cells, proliferation, migration, cytokine array, peripheral nervous system

## Abstract

The peripheral nervous system has remarkable regenerative capabilities. Schwann cells and fibroblasts are known to play crucial roles in these processes. In this study, we delineated the differential effects of peripheral nerve fibroblasts and cardiac fibroblasts on Schwann cells. We found that peripheral nerve fibroblasts significantly promoted Schwann cell proliferation and migration compared with cardiac fibroblasts. The cytokine array results identified 32 of 67 proteins that were considered differentially expressed in peripheral nerve fibroblasts versus cardiac fibroblasts. Among them, 25 were significantly upregulated in peripheral nerve fibroblasts compared with cardiac fibroblasts. Activin A, the protein with the greatest differential expression, clearly co-localized with fibroblasts in the *in vivo* sciatic never injury rat model. *In vitro* experiments proved that activin A secreted from nerve fibroblasts is the key factor responsible for boosting proliferation and migration of Schwann cells through ALK4, ALK5, and ALK7. Overall, these findings suggest that peripheral nerve fibroblasts and cardiac fibroblasts exhibit different patterns of cytokine secretion and activin A secreted from peripheral nerve fibroblasts can promote the proliferation and migration of Schwann cells.

## Introduction

The peripheral nervous system has a remarkable regeneration capacity relative to the central nervous system, which is dependent on the strength of Schwann cells (SCs) and fibroblasts (Fbs). SCs, Fbs, axons, as well as a small number of macrophages, neutrophils, and vascular endothelial cells, are the components of peripheral nerve fibers (Dreesmann et al., 2009). Nerve fibroblasts (N-Fbs), as a fundamental constituent of the epineurium, perineurium, and endoneurium, provide an extracellular microenvironment that contributes to neuronal regeneration (Sorrell and Caplan, 2009). Single-cell transcriptome data analysis has shown that N-Fbs can be sub-clustered into epineurial Fbs, perineurial Fbs, and endoneurial Fbs—after peripheral nerve injury, SCs and endoneurial Fbs are the most important cell types for promoting nerve regeneration (Chen et al., 2021). After peripheral nerve transection, N-Fbs can regulate the collective migration of SCs to guide neurite growth within the nerve bridge formation and accelerate regeneration. Previous *in vitro* studies have suggested that N-Fbs regulate SC migration by factors from Fb conditional medium (CM) (van Neerven et al., 2013), such as neuregulin-1b1 (Dreesmann et al., 2009). In addition, Fbs were proved to secrete tenascin-C, which accelerates SC migration through the integrin β1 signaling pathway (Zhang et al., 2016). When N-Fbs are co-cultured with SCs, N-Fbs cause some early instances of peripheral nerve regeneration, including basal laminin deposition and SC elongation (Obremski et al., 1993). Co-transplantation of Fbs with SCs has been found to facilitate nerve regeneration and functional recovery as well as significantly increase the number of regenerated axons, their mean diameters, and the thickness of the myelin sheath of such axons (Wang et al., 2017). Important progress was made when ephrin-B/EphB2 signaling between Fbs and SCs was revealed to result in the directional collective cell migration of SCs to guide regrowing axons across the wound (Parrinello et al., 2010). These findings suggest that Fbs promote SC migration and neuronal growth by secreting proteins.

Activin A, a member of the transforming growth factor β (TGFβ) superfamily, plays an important role in cell proliferation, migration, differentiation, and immune regulation. Researchers have claimed that activin A secreted from human apical papilla stem cells can decrease neuroinflammation and stimulate oligodendrocyte progenitor differentiation (De Berdt et al., 2018). *In vitro* studies have demonstrated that activin A stimulation in the presence of IL-34 and cholesterol promotes microglia survival (Bohlen et al., 2017). In an Alzheimer’s disease model, activin A secreted by human mesenchymal stem cells induced neuronal development and neurite outgrowth (Park et al., 2016). Activin signaling through Smad2/3 is a conserved neural regenerative pathway activated in the injured nervous system (Morianos et al., 2019). Activin A signals through two type I and two type II receptors which, upon ligand binding, activate their kinase activity and phosphorylate the Smad2 and Smad3 intracellular signaling mediators that form a complex with Smad4, which subsequently translocates to the nucleus and activates or silences gene expression (Morianos et al., 2019). Our cytokine array results show that activin A expression was much higher in N-Fbs compared with cardiac fibroblasts (C-Fbs). However, the biological functions of activin A on SCs have not been reported.

In this study, we found proliferating and migrating SC levels increased significantly when co-cultured with N-Fbs compared with C-Fbs. *In vitro* experiments demonstrated that activin A secreted from Fbs could promote SC proliferation and migration through its type I and type II receptors.

## Materials and Methods

### Primary Schwann Cell and Fibroblast Cultures

Rat neonatal N-Fbs and SCs were isolated and cultured as previously described. Sciatic nerves were dissected from neonatal (day 1) Sprague–Dawley rats, finely cut into ∼4-mm segments, and then incubated at 37°C in collagenase type XI (Sigma) for 30 min, followed by another incubation in 0.25% (w/v) trypsin/EDTA (Gibco) for 10 min. Enzymes were inactivated, and the suspension was centrifuged. The cell pellet was resuspended in DMEM (Corning) with 10% FBS and 1% antibiotics (penicillin/streptomycin, Gibco), hereinafter referred to as culture medium, filtered through a 400-mesh filter to obtain a single-cell suspension, and then plated in 60-mm Petri dishes. Due to the limited number of isolated primary cells per neonatal rat, Fbs and SCs isolated from per eight rats were used in one independent experiment.

When the cultures reached 90% confluence, SCs and N-Fbs were isolated by differential digestion and differential adherence (He et al., 2020). Specifically, SCs were collected after trypsinization with 0.25% trypsin for 8-10 s at room temperature, mixed with culture medium to stop the digestion, gently blown with a pipette to detach, and then centrifuged. SCs were inoculated in an uncoated Petri dish and cultured with SC culture medium (SCCM, DMEM supplemented with 10% FBS, 2 μM forskolin, 10 ng/ml HRG, and 1% penicillin/streptomycin) at 37°C for 30-45 min. During this time, the small amount of N-Fbs which were digested with SCs attached to the dish. SCs in the supernatant were transferred to another poly-L-lysine (PLL)-coated Petri dish and cultured with SCCM.

After removing the SCs, the remaining N-Fbs were washed, digested for 2 min at 37°C, blown, collected after stopping digestion by adding culture medium, and then centrifuged. N-Fbs were inoculated in an uncoated Petri dish and attached to the bottom after 30-45 min. N-Fb culture was continued in fresh culture medium after the supernatant, including a few SCs, was discarded. P1 SCs and N-Fbs were passaged until they reached 90% confluence and purified by differential digestion and differential adherence as described above.

Rat neonatal C-Fbs were isolated and cultured as previously described (Thum et al., 2008). In brief, the ventricular muscles from 1-day-old Sprague–Dawley rats were finely minced into 1–2 mm^2^ pieces and digested with trypsin for 30-40 min at 37°C under constant stirring. The collected primary cells were passed through a 400-mesh filter, inoculated in Petri dishes, and incubated for approximately 45 min at 37°C in a CO_2_ incubator. During this time, only the Fbs became attached to the Petri dish. The adherent C-Fbs were washed several times and then cultured in culture medium.

The purities of the isolated Fbs and SCs were determined by staining antibodies directed against the fibroblast-specific antigen CD90 and glia-specific antigen GFAP.

### Immunocytochemical Staining

**FIGURE 1:**
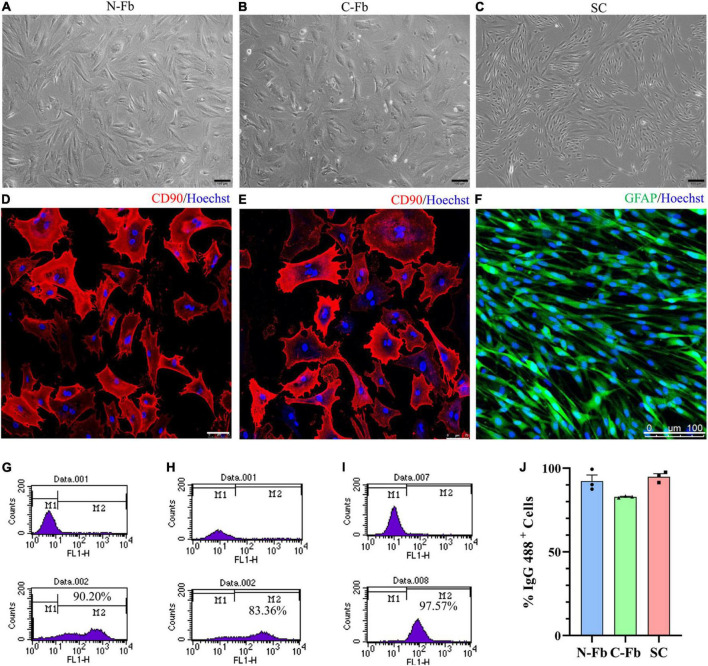
Cell characterization and purification. Phase-contrast micrographs of live primary cultured N-Fbs **(A)**, cardiac fibroblasts **(B)**, Schwann cells **(C)**. Scale bar, 100 μm. Immunofluorescence for fibroblast marker CD90 in purified N-Fbs **(D)**, C-Fbs **(E)**, and SC marker GFAP in purified SCs **(F)**. Scale bar: **(D,E)** 75 μm; **(F)** 100 μm. Representative figures of flow cytometry analysis of purified N-Fbs **(G)**, C-Fbs **(H)**, SCs **(I)**. The upper graphics are control groups, and the lower graphics are experimental groups. M1/M2 stood for the area under the curve in upper/lower graphics; the percentage of M2 relative to M1 + M2 was the percentage of IgG 488-positive cells. **(J)** The purity of N-Fbs, C-Fbs, and SCs (three independent experiments).

Immunocytochemical staining was performed after differential digestion and differential adherence of Fbs and SCs. Fbs were fixed with 4% paraformaldehyde, blocked with blocking buffer (0.01 M PBS containing 5% goat serum) for 45 min at 37°C after washing, incubated overnight at 4°C with primary antibodies, including mouse monoclonal anti-CD90 (1:1000, Cat# ab225, Abcam) and goat anti-activin A (10 μg/mL, Cat# A1594-0.1MG, Sigma-Aldrich), and reacted with the following secondary antibodies: Alexa Fluor 594 goat anti-mouse IgG (1:400, Cat# ab150116, Abcam) (Figure 1) and 488 goat anti-mouse IgG (1:400, Cat# ab150113, Abcam) and Cy3 donkey anti-goat IgG (1:400, Cat# ab6949, Abcam). Finally, the Fbs were subject to Hoechst 33258 (1:500, Cat# C1011, Beyotime Biotechnology) counterstaining at room temperature for 15 min.

After washing, SCs were fixed and blocked with blocking buffer (0.1% Triton X-100 in 0.01M PBS containing 5% goat serum), incubated overnight at 4°C with chicken anti-glial fibrillary acidic protein (anti-GFAP) primary antibody (1:500, Cat# ab254083, Abcam), and then incubated with Alexa Fluor 488 goat anti-chicken IgY (1:400, Cat# ab150169, Abcam). The SCs were then subject to Hoechst 33258 (1:500) counterstaining at room temperature for 15 min. Images were captured using a confocal laser scanning microscope (TCS SP5, Leica Microsystems, Wetzlar, Germany).

### Flow Cytometric Analysis

To further provide quantitative evidence of cell purity, cells were analyzed by flow cytometry. Over 1 × 10^6^ cells were collected at 48 h after the second passage for both the control and test groups. Test group Fbs and SCs were washed with PBS three times prior to overnight incubation at 4°C with the following primary antibodies: mouse anti-CD90 (1:500, Cat# ab23894, Abcam) for Fbs and rabbit anti-S100 (1:100, Cat# SAB5500172, Sigma) for SCs. In the control groups, cells were incubated with PBS under the same conditions. Both groups of Fbs were labeled with secondary antibody donkey anti-mouse IgG H&L 488 (1:500, Cat# ab150105, Abcam) and both groups of SCs with secondary antibody goat anti-rabbit IgG H&L 488 (1:500, Cat# ab150077, Abcam) for 2 h at room temperature. Samples of each group were then washed and resuspended in PBS. IgG 488-positive cells were detected using BD FACSCalibur and CellQuest software (BD Bioscience, San Jose, CA, United States), the percentage of which was calculated.

### Fb/SC Co-culture and Schwann Cell Migration in a Transwell System

To co-culture SCs with Fbs, 4 × 10^4^ cells/well N-Fbs/C-Fbs were suspended in DMEM and placed in the upper compartment of a 6.5-mm Transwell chamber with an 8.0-μm pore (Corning). 4 × 10^4^ cells/well SCs suspended in DMEM with 1% FBS and 1% antibiotics (penicillin/streptomycin), referred to as the serum deprivation medium, were placed in a poly-L-lysine-coated bottom chamber. After incubation for 24 h at 37°C, proliferated SCs stained by an EdU assay were counted and calculated under an optical microscope.

For the SC migration experiment, 1 × 10^5^ cells/well Fbs suspended in culture medium were added to the bottom chamber. After 24 h in the 37°C incubator, 4 × 10^4^ cells/well SCs suspended in DMEM were placed in the upper compartment of a 6.5-mm Transwell chamber with an 8.0-μm pore. At 6 h, 12 h, and 24 h, migrated SCs with crystal violet staining were observed and counted under the optical microscope.

### EdU Assay

EdU assays were performed on SCs after a 24-h incubation in 24/96-well plates according to the manufacturer’s instruction using the Cell-Light EdU Apollo 567 *In Vitro* Imaging Kit (RiboBio). SCs were labeled with EdU, fixed with 4% paraformaldehyde, and stained with Apollo 567 and Hoechst 33342. Cells were counted under an optical microscope, and the percentage of EdU-positive cells relative to the total amount of cells was calculated.

### Crystal Violet Staining

After fixing with 4% paraformaldehyde and staining with crystal violet staining solution (Beyotime) for 22 min at room temperature, the cells left in the upper chamber of the transwell system were gently wiped with a cotton swab. The migrated cells attached to the bottom side of the transwell system were carefully rinsed and then photographed by an inverted microscope. These cells were counted, and their percentage was calculated.

### G-Series Rat Cytokine Array 67

1 × 10^5^ cells/well purified N-Fbs/C-Fbs were seeded in a 24-well plate. After 24 h, the medium was replaced by co-culture medium (500 μl per well). After another 36-h incubation at 37°C, the supernatant was filtered through a 0.22-μm filter and collected, hereinafter known as the N-Fb CM/C-Fb CM.

One hundred microliters of sample (N-Fb/C-Fb CM) diluent was added into each well of the array-specific glass slides, incubated at room temperature for 30 min to block the slides, and then decanted. Another 100 μl of sample diluent was added to each well, incubated at room temperature for 1-2 h, and then decanted. Slides were washed five times (five min each) with 150 μl of 1× wash buffer I at room temperature with gentle shaking in the Wellwash Versa (Thermo Scientific) and were then subject to the previous wash steps two times with 1× wash buffer II.

To reconstitute the detection antibody, 1.4 ml of sample diluent was added to the tube. Eighty microliters of the detection antibody cocktail was added to each well and then incubated at room temperature for 1-2 h. The slides were then washed again as described above.

After briefly being spun down, 1.4 ml of sample diluent was added to a Cy3 equivalent dye-conjugated streptavidin tube and gently mixed. Eighty microliters of the detection antibody cocktail was added to each well and incubated in a dark room at room temperature for 1 h. The slides were then washed again as described above.

Slides went through fluorescence detection on the InnoScan 300 Microarray Scanner (Innopsys) with a wavelength of 532 nm and a resolution of 10 μm. Data were extracted using microarray analysis software (GenePix). Results included (log2) fold changes for each protein and for each contrast individually. Differentially expressed proteins (DEPs) were defined as those with a foldchange greater than 1.2 or less than 0.83 (an absolute value of logFC > 0.263).

### Quantitative Reverse Transcription Polymerase Chain Reaction

Total RNA was isolated from cells using TRIzol reagent (QIAGEN), and cDNA was obtained using an Omniscript RT kit (QIAGEN) under the guide of the manufacturer’s instructions. qRT-PCR was performed with SYBR Premix (QIAGEN) on the BIO-RAD system (BIO-RAD-96CFX) following the standard procedure. Stem-loop RT primers (QIAGEN) were used to quantify the expression of miRNA, and the expression of GAPDH was used as a reference. The specific protocol was based on the manufacturer’s instructions, and miRNA primers were synthesized by RiboBio. The results were analyzed by the 2^–ΔΔCt^ method.

### ELISA

ELISA kits (CUSABIO) were used to detect designated protein (activin A, eotaxin, and Csf2) concentration. First, blank wells were set in a 96-well plate. One hundred microliters of standard or sample (N-Fb/C-Fb CM) was added per well, covered with an adhesive strip, and incubated for 2 h at 37°C. After removing the liquid in each well, 100 μl of biotin antibody (1×) was added to each well. The wells were subsequently covered and incubated for 1 h at 37°C. Each well was decanted and washed three times with 200 μl of wash buffer. One hundred microliters of HRP–avidin (1×) was added per well. Once again, the wells were covered and incubated for 1 h at 37°C. After five washes, 90 μl of TMB substrate was added to each well. The wells were incubated for 15-30 min at 37°C but protected from light. Then, 50 μl of stop solution was added to each well and mixed by gently tapping the plate. The optical density of each well was determined within 5 min using a microplate reader set to 450 nm.

### Immunohistochemistry

The expression of activin A was observed after sciatic nerve transection in male SD rats, to avoid the interference of estrogen. Adult male Sprague–Dawley rats weighing 180 g were anesthetized with intraperitoneal sodium pentobarbital (30 mg/kg) before transection of the right sciatic nerve. At day 7 after nerve transection, deeply anesthetized rats were perfused sequentially with normal saline and 4% paraformaldehyde. The sciatic nerve was removed, fixed, and dehydrated in a series of graded sucrose solutions. The nerve was embedded with an optimal cutting temperature compound, frozen in freezing microtome, and then cut into transverse sections at intervals of 12 μm. Nerve sections were blocked with blocking buffer (0.01M PBS containing 0.1% Triton X-100 and 10% goat serum) for 45 min at 37°C, then incubated overnight at 4°C with primary antibodies, including mouse monoclonal anti-CD90 (1:400) and goat anti-activin A (10 μg/mL), and correspondingly reacted with the secondary antibodies for 2 h in dark at room temperature: Alexa Fluor 488 goat anti-mouse IgG (1:500) Cy3 donkey anti-goat IgG (1:500). The samples were subject to Hoechst 33258 (1:500) counterstaining at room temperature for 10 min. Images was captured under a confocal laser scanning microscope.

### Schwann Cell Proliferation With Fb Culture Medium, Activin A, and Inhibitor

Schwann cells were seeded in a density of 1 × 10^4^ cells/well in 96-well plate. When SCs attached to the bottom (4-6 h), the medium was replaced by Fb-conditioned medium or serum deprivation medium with 0 ng/mL (control group), 10 ng/mL, 20 ng/mL, and 40 ng/mL activin A (R&D Systems). The wells were then incubated for 24 h. Proliferated SCs stained by an EdU assay were counted and calculated under the optical microscope.

The effect of SB-431542 (Selleck), an inhibitor of activin receptor-like kinase 4 (ALK4), ALK5, and ALK7, on SC proliferation was tested using a similar method as above. After 4-6 h, the medium was replaced by solely serum deprivation medium (control group) or serum deprivation medium with 10 μM SB-431542, N-Fb CM, N-Fb CM plus 10 μM SB-431542, 40 ng/mL activin A, or 40 ng/mL activin A plus 10 μM SB-431542, respectively. Proliferated SCs were stained by EdU assay 24 h later.

### Schwann Cell Migration With Fb Culture Medium, Activin A, and Inhibitor

Schwann cells were seeded in a density of 3 × 10^4^ cells per well in a Culture-Insert 2-well (Ibidi) in a PLL-coated Petri dish. When SCs attached to the bottom (after 4-6 h), the medium was replaced by Fb-conditioned medium or serum deprivation medium with 0 ng/mL (control group), 10 ng/mL, 20 ng/mL, or 40 ng/mL activin A. After incubating for 12 h, corresponding medium was added outside the chambers in the Petri dish of each group, and then, Culture-Inserts were removed to create a cell-free gap for wound healing. SCs were immediately monitored by phase-contrast microscopy. Optical microscopic images were taken at 0 h and 8 h post-gap creation. Measurements of cell-free areas at different time points were taken using ImageJ software.

The effect of SB-431542 on SC migration was tested by a similar method as above. After 4-6 h, the medium was replaced by serum deprivation medium (control group) or serum deprivation medium with 10 μM SB-431542, N-Fb CM, N-Fb CM plus 10 μM SB-431542, 40 ng/mL activin A, or 40 ng/mL activin A plus 10 μM SB-431542, respectively. After 12 h pretreatment, optical microscopic images were taken at 0 h and 8 h post-gap creation.

### Schwann Cell Proliferation With Fb Culture Medium, Activin A, and Activin A-Neutralizing Antibody

Schwann cells were seeded in a density of 1 × 10^4^ cells/well in a 96-well plate. The effect of neutralizing antibody goat anti-activin A (1 μg/mL, Cat# A1594-0.1MG, Sigma-Aldrich) on SC proliferation was tested using the same procedure as described above. After 4-6 h, the medium was replaced by solely serum deprivation medium (control group) or serum deprivation medium with 1 μg/mL activin A-neutralizing antibody, N-Fb CM, N-Fb CM plus 1 μg/mL activin A-neutralizing antibody, 40 ng/mL activin A, or 40 ng/mL activin A plus 1 μg/mL activin A-neutralizing antibody, respectively. Proliferated SCs were stained by EdU assay 24 h later.

### Schwann Cell Migration With Fb Culture Medium, Activin A, and Activin A-Neutralizing Antibody

Schwann cells were seeded in a density of 3 × 10^4^ cells per well in a Culture-Insert 2-well in a PLL-coated Petri dish. The effect of activin A-neutralizing antibody on SC migration was tested using the same procedure as described above. After 4-6 h, the medium was replaced by serum deprivation medium (control group) or serum deprivation medium with 1 μg/mL activin A-neutralizing antibody, N-Fb CM, N-Fb CM plus 1 μg/mL activin A-neutralizing antibody, 40 ng/mL activin A, or 40 ng/mL activin A plus 1 μg/mL activin A-neutralizing antibody, respectively. After 12 h pretreatment, optical microscopic images were captured at 0 and 8 h post-gap creation.

### Statistical Analysis

All quantitative data were presented as mean ± SEM from at least three independent experiments made with biological uniplicates. Statistical analysis was conducted using GraphPad Prism 8.0 software (GraphPad Software, San Diego, CA, United States). Comparisons between groups were assessed using unpaired Student’s *t*-test (two-tailed), one-way analysis of variance (ANOVA), two-way ANOVA (followed by Tukey’s *post hoc* test). Significance for all tests was defined as *p < 0.05, ^**^p < 0.01, ^***^p < 0.001, and ^****^p < 0.0001.

## Results

### Cell Characterization and Purification

After the primary culture at two to three passages, purified Fbs and SCs were observed under a phase-contrast microscope (Figures 1A–C). The morphology of the N-Fbs and C-Fbs showed a typical large, flat, and elongated shape; that of the SCs presented a typical bipolar spindle shape. Immunofluorescence staining results demonstrated the positive expression of fibroblast-associated marker proteins CD90 (Figures 1D,E) and SC-associated marker proteins GFAP (Figure 1F). The flow cytometry results showed that these cultures contained almost exclusively primary fibroblasts or SCs (Figures 1G–I). N-Fbs and C-Fbs that were Thy-1 positive (CD90^+^) comprized an average of 92 and 83% of the total cells at the second passage, respectively. SCs that were S100 positive (S100^+^) accounted for 95% of the total cell numbers.

### N-Fbs Facilitate Schwann Cells Migration and Proliferation *in vitro*

**FIGURE 2:**
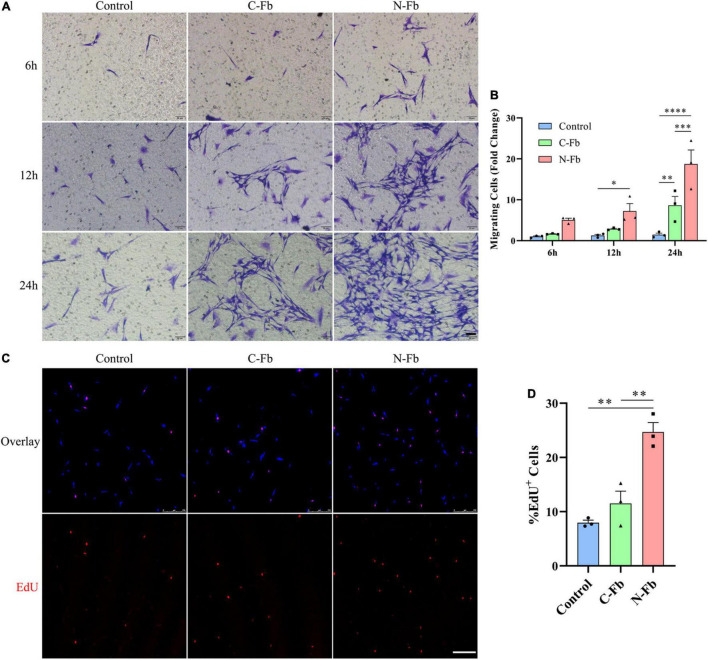
N-Fbs facilitates Schwann cells migration and proliferation *in vitro*. **(A)** Phase-contrast micrographs of crystal violet-labeled migrated SCs co-cultured with blank control, C-Fbs, and N-Fbs in transwell for 6 h, 12 h, and 24 h. Scale bar, 50 μm. **(B)** Migrating SCs quantification (three independent experiments). Statistical significance is indicated as *p < 0.05 N-Fb-12h vs. Control-12h, ^**^p < 0.01 C-Fb-24h vs. Control-24h, ^***^p < 0.001 N-Fb-24h vs. C-Fb-24h, ^****^p < 0.0001 N-Fb-24h vs. Control-24h (two-way analysis of variance followed by Tukey’s *post hoc* test). **(C)** EdU^+^ SCs (red) were labeled after 24-h co-culture with N-Fbs and C-Fbs, separately. Scale bar, 75 μm. **(D)** Quantification of the proportion of EdU^+^ SCs (three independent experiments). Statistical significance is indicated as ^**^p < 0.01 (one-way analysis of variance followed by Tukey’s *post hoc* test).

The potential role of N-Fbs in modulating the migration and proliferation abilities of SCs was investigated by transwell migration and EdU assays. Transwell migration assays indicated that SCs co-cultured with N-Fbs possessed a higher migration ability compared with SCs co-cultured with C-Fbs or blank control (Figures 2A,B, respectively). Specifically, after co-culture for 6 h, the number of SCs migrated to the underside of the transwell in the N-Fb group increased relative to the control group, although the difference was not statistically significant. After 12 h, N-Fb significantly promoted the migration of SCs (N-Fb-12h vs. Control-12h, *p* < 0.05). While the incubation time extended to 24 h, the number of migrating SCs in the C-Fb group increased (C-Fb-24h vs. Control-24h, *p* < 0.01), which in the N-Fb group increased significantly compared with the former (N-Fb-24h vs. C-Fb-24h, *p* < 0.001).

EdU assays revealed that the cell proliferation of SCs co-cultured with N-Fbs for 24 h was significantly higher than that of SCs co-cultured with C-Fbs or blank control (Figures 2C,D, respectively). SCs co-cultured with C-Fbs showed slightly higher proliferation than the blank control, but no significant difference was observed here.

### Differentially Expressed Proteins in N-Fb CM vs. C-Fb CM

**FIGURE 3:**
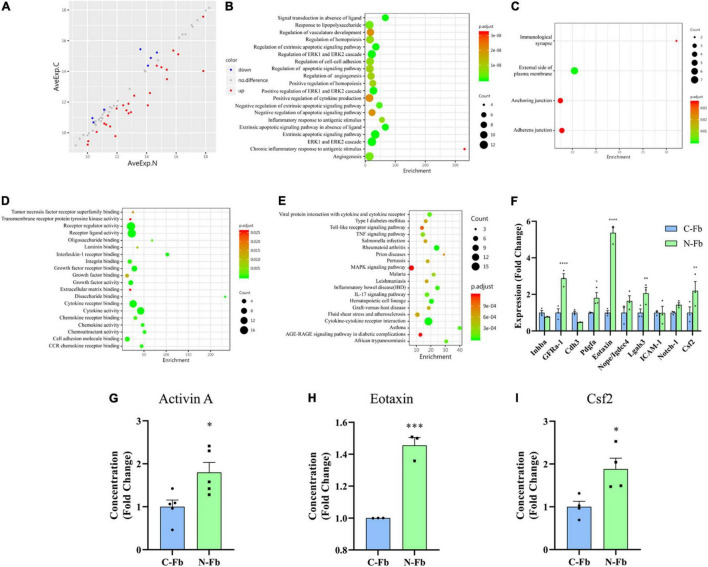
Differentially expressed proteins in N-Fb CM vs. C-Fb CM. **(A)** Scatter plot of 32 differentially expressed proteins in N-Fb vs. C-Fb-conditioned medium with Log2-foldchange greater than 1.2 or less than 0.83. Red represents the upregulation, while blue represents the downregulation, and gray resembles no difference. Protein function annotation Gene Ontology (GO) and KEGG pathway were analyzed. GO analysis included three subtypes: **(B)** BP, **(C)** MF, and **(D)** CC. **(E)** KEGG pathway results reveal associated gene functions, linking genomic information with higher-order functional information. **(F)** q-PCR results of top ten differentially expressed proteins in N-Fb vs. C-Fb (three independent experiments). Statistical significance is indicated as *p < 0.05, ^**^p < 0.01, ^****^p < 0.0001 (unpaired Student’s *t*-test). ELISA results of activin A **(G)** (five independent experiments), Eotaxin **(H)** (three independent experiments), and Csf2 **(I)** (four independent experiments). Statistical significance is indicated as *p < 0.05, ^***^p < 0.001 (unpaired Student’s *t*-test).

As our results suggest, N-Fb-derived proteins have the potential of promoting SC proliferation and migration. The proteins that are differentially expressed in N-Fb CM and C-Fb CM could be the key to revealing the mechanisms behind this. GSR-CAA-67 was performed on N-Fb CM and C-Fb CM collected after a 36-h incubation. The results identified 25 DEPs with a log2-foldchange greater than 1.2 (red) and seven DEPs with a log2-foldchange less than 0.83 (blue) among the 67 proteins that were tested (Figure 3A and Supplementary Table 1). Activin A had the largest log2-foldchange among the 25 DEPs more highly expressed in N-Fb CM. Protein function annotation Gene Ontology (GO) and KEGG pathways were subsequently analyzed. GO analysis included three subtypes: biological process (BP, Figure 3B), cellular component (CC, Figure 3C), and molecular function (MF, Figure 3D). Noteworthy, more DEPs were enriched in regulation of cell-cell adhesion, positive regulation of ERK1 and ERK2 cascade, positive regulation of cytokine production, negative regulation of apoptotic signaling pathway, angiogenesis, receptor regulator activity, receptor ligand activity, cytokine receptor binding, and cytokine activity. The KEGG database was used to systematically analyze gene function, linking genomic information to higher-order functional information (Figure 3E). Most DEPs were enriched in cytokine–cytokine receptor interaction.

To verify the results from GSR-CAA, the top ten DEPs ranked by logFC value were analyzed using q-PCR assays. Among these DEPs, GFRa-1, Pdgfa, Ccl11, Lgals3, and Csf2 showed significant differential expression among N-Fbs versus C-Fbs (Figure 3F). Meanwhile, ELISA results demonstrated that three DEPs (activin A, gene name: Inhba; eotaxin, gene name: Ccl11; Csf2) had significantly higher expression in N-Fbs compared with C-Fbs (Figures 3G–I). The most significant DEP, activin A, was chosen for further investigation.

### Localization and Expression of Activin A in N-Fbs *in vitro* and Rat Sciatic Nerve Injury Model *in vivo*

**FIGURE 4:**
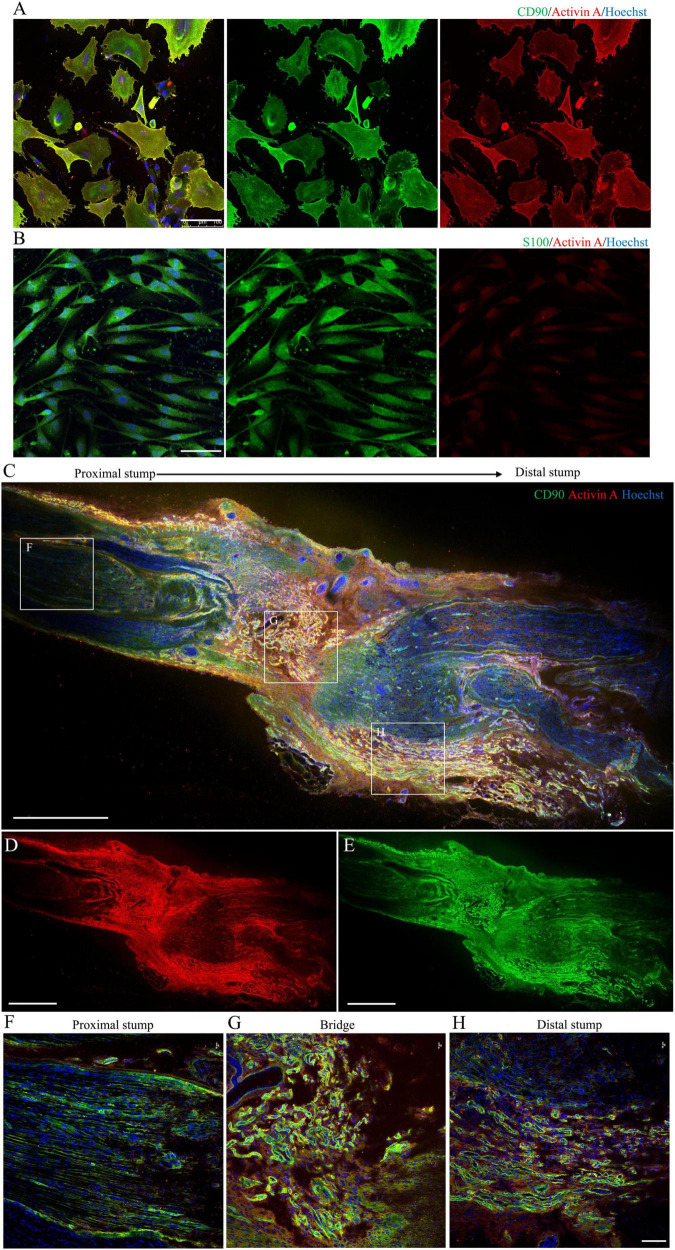
Localization and expression of activin A in N-Fbs *in vitro* and rat sciatic nerve injury model *in vivo*. **(A)** Immunofluorescence for activin A and fibroblast marker CD90 in purified N-Fbs. Scale bar, 100 μm. **(B)** Immunofluorescence for activin A and SC marker S100 in purified SCs. Scale bar, 100 μm. Expression of activin A in rat sciatic nerve injury model *in vivo*. **(C–E)** Immunofluorescence images of activin A **(D)** and CD90 **(E)** in sciatic nerve, proximal to distal. Scale bar, 1000 μm. **(F–H)** Magnified images of the regions demarcated by white boxes in **(C)**. Scale bar, 100 μm.

Immunofluorescence imaging showed that activin A was mainly located in the cytoplasm and cytomembrane of purified N-Fb *in vitro* (Figure 4A). As indicated by the staining, a large number of Fbs aggregated on the injured site, forming a nerve bridge connecting the proximal and distal stumps (Figures 4C–E). As shown in magnified images, activin A signals predominantly co-located with Fbs in the bridge region (Figure 4G) and distal regenerating region (Figure 4H). In the proximal stump, very little activin A signals were detected except for the outer edge of nerve (Figure 4F). On the other hand, the expression of activin A in SC was hardly detected by immunocytochemical staining (Figure 4B).

### Activin A Facilitates Schwann Cell Proliferation and Migration *in vitro*

**FIGURE 5:**
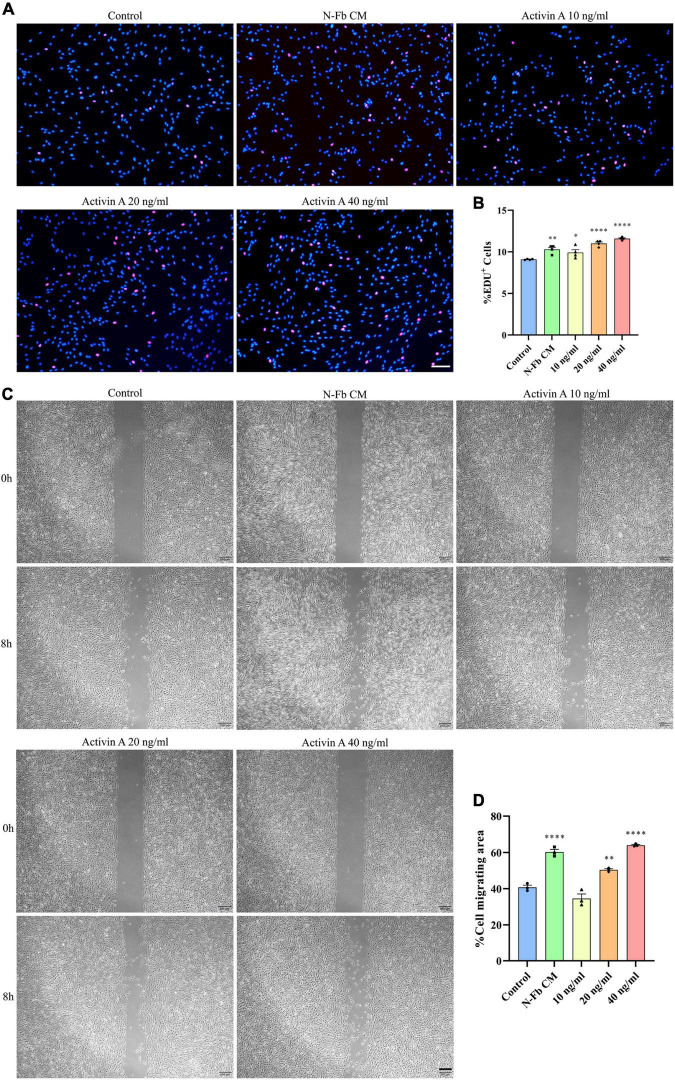
Activin A facilitates Schwann cells proliferation and migration *in vitro*. **(A)** EdU^+^ SCs (red) were labeled after 24 h cultured with N-Fb CM and activin A (10-40 ng/ml), separately. Scale bar, 100 μm. **(B)** Quantification of the proportion of EdU^+^ SCs (4 independent experiments). Statistical significance is indicated as *p < 0.05, ^**^p < 0.01, ^****^p < 0.0001 (one-way analysis of variance followed by Tukey’s *post hoc* test). **(C)** Phase-contrast micrographs of SCs in the cell-free gap at 0 h and 8 h (after 12 h pretreatment) with blank control, activin A (10-40 ng/ml), separately. Scale bar, 200 μm. **(D)** Quantification of the SC migrating area percentage relative to the cell-free area at 0 h (three independent experiments). Statistical significance is indicated as ^**^p < 0.01, ^****^p < 0.0001 (one-way analysis of variance followed by Tukey’s *post hoc* test).

To determine whether activin A is the factor present in N-Fb CM critical for promoting SC proliferation, EdU assays were conducted. SCs cultured for 24 h with N-Fb CM or different concentrations of activin A showed different proliferation capabilities (Figure 5A). SCs in the N-Fb CM group demonstrated significantly higher proliferation rates (Figure 5B). Among the activin A groups, 40 ng/mL was proven to be the most effective concentration for facilitating SC proliferation, while 20 ng/mL group also had significantly higher proliferation rates compared with the N-Fb CM group. The effect of the activin A was dose related.

Meanwhile, wound healing assays were performed to test the capability of activin A to promote SC migration. After 12 h pretreatment with N-Fb CM or different concentrations of activin A, the migration rate of SCs in 8 h significantly increased in the N-Fb CM group compared with the control group (Figures 5C,D). Activin A groups showed a similar trend to the results of proliferation rates in EdU assays. 40 ng/mL activin A promoted the migration of Schwann cells significantly, while 20 ng/mL group also had significantly higher migration rates compared with the control group, but not as well as N-Fb CM group.

### SB-431542 Inhibits the Proliferation and Migration Promotion Effects of N-Fb CM and Activin A on Schwann Cells *in vitro*

**FIGURE 6:**
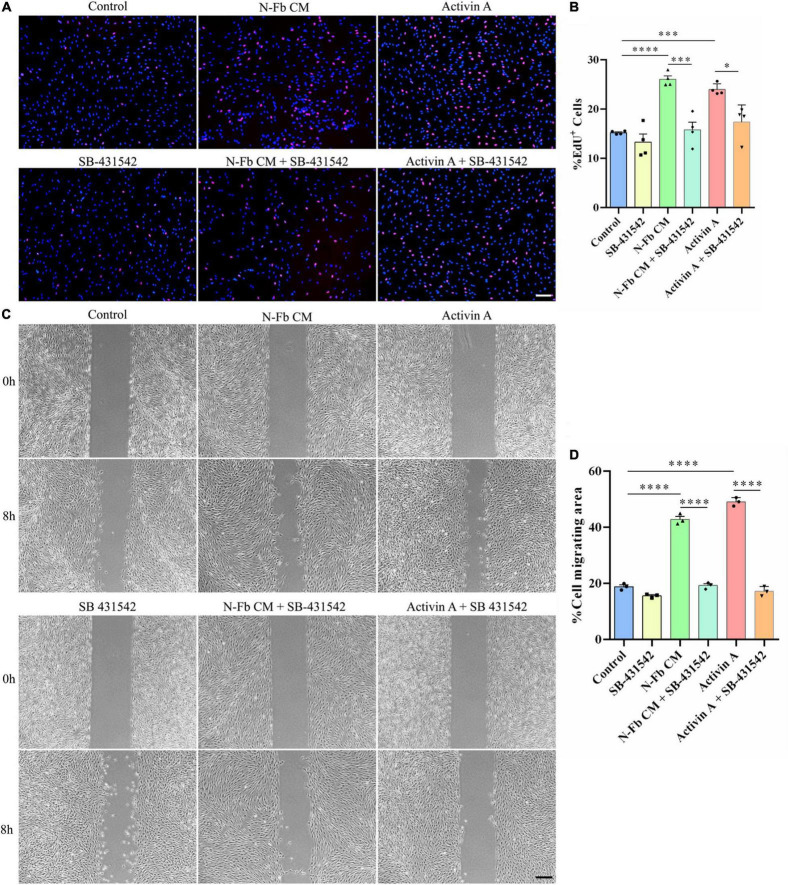
SB-431542 inhibits the proliferation and migration promotion effects of N-Fb CM and activin A on Schwann cells *in vitro*. **(A)** EdU^+^ SCs (red) were labeled after 24-h culture. Scale bar, 100 μm. **(B)** Quantification of the proportion of EdU^+^ SCs. Statistical significance is indicated as *p < 0.05, ^***^p < 0.001, ^****^p < 0.0001 (four independent experiments, one-way analysis of variance followed by Tukey’s *post hoc* test). **(C)** Phase-contrast micrographs of SCs in the cell-free gap at 0 h and 8 h (after 12 h pretreatment). Scale bar, 200 μm. **(D)** Quantification of the SC migrating area percentage relative to the cell-free area at 0 h (three independent experiments). Statistical significance is indicated as ^****^p < 0.0001 (one-way analysis of variance followed by Tukey’s *post hoc* test).

SB-431542 is a selective inhibitor of TGFβ superfamily type I ALK receptors ALK4, ALK5, and ALK7. To verify whether the stimulatory effects of N-Fb CM and activin A on SCs were through ALK signaling, EdU assays and wound healing methods were performed. N-Fb CM and activin A significantly accelerated the proliferation of SCs. The effect of N-Fb CM and activin A on SC proliferation was significantly abolished by SB-431542 (10 μM) after incubation for 24 h (Figures 6A,B). N-Fb CM and activin A significantly increased the migration rate of SCs. The increase in SC migration rate caused by N-Fb CM and activin A was also significantly abolished by SB-431542 after 12 h pretreatment and 8-h observation (Figures 6C,D). To briefly recapitulate, the inhibition of ALK receptors abolished the proliferation and migration promotion effects of N-Fb CM and activin A on SCs *in vitro*.

### Activin A-Neutralizing Antibody Inhibits the Proliferation and Migration Promotion Effects of N-Fb CM and Activin A on Schwann Cells *in vitro*

**FIGURE 7:**
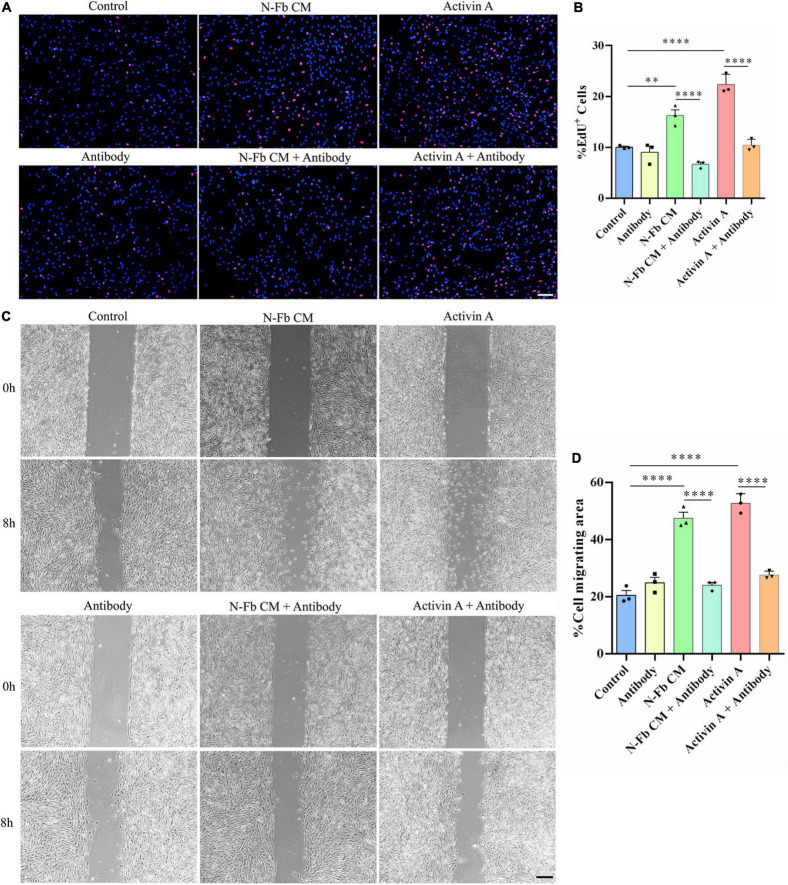
Activin A-neutralizing antibody inhibits the proliferation and migration promotion effects of N-Fb CM and activin A on Schwann cells *in vitro*. **(A)** EdU^+^ SCs (red) were labeled after 24-h culture. Scale bar, 100 μm. **(B)** Quantification of the proportion of EdU^+^ SCs. Statistical significance is indicated as ^**^p < 0.01, ^****^p < 0.0001 (three independent experiments, one-way analysis of variance followed by Tukey’s *post hoc* test). **(C)** Phase-contrast micrographs of SCs in the cell-free gap at 0 h and 8 h (after 12 h pretreatment). Scale bar, 200 μm. **(D)** Quantification of the SC migrating area percentage relative to the cell-free area at 0 h (three independent experiments). Statistical significance is indicated as ^****^p < 0.0001 (one-way analysis of variance followed by Tukey’s *post hoc* test).

Since SB-431542 is a TGFβ superfamily receptor inhibitor, which blocks the signaling of not only activin A, but also other members of the TGFβ family, activin A-neutralizing antibody was used to conclude activin A was responsible for the proliferative and migratory effects of SCs. The results of EdU assays and wound healing methods suggested a similar trend. The effects of N-Fb CM and activin A on SC proliferation (Figures 7A,B) and migration (Figures 7C,D) were significantly abolished by activin A-neutralizing antibody. Therefore, the promotion of proliferative and migratory effects on SCs was attributed to activin A, rather than other members of the TGFβ family.

## Discussion

Fbs can be classified into subtypes based on the tissue sources in which they are located. Certain subtypes of Fbs are known to interact with other cells derived from the same tissue—for instance, epithelial Fbs with epithelial cells, and peripheral nerve Fbs with SCs (Krieg et al., 2007; Zhang et al., 2016; Kadota et al., 2020). Fbs and SCs were revealed to promote nerve regeneration through direct contact cell–cell signaling, secreted factors, and ECM interactions (Peng et al., 2020). In our study, the SCs were co-cultured with C-Fbs and N-Fbs in transwell system without direct membrane contact (Figure 2). Various factors, including activin A, are continuously secreted from the Fbs during the co-culture, thus suggesting that there could be a mutual crosstalk between SCs and Fbs, while in the CM there were only the factors already secreted from fibroblasts even in the absence of SCs (Figure 5). On the other hand, condition of cells may vary from batch to batch. Still, in Figures 5, 6, the trend is consistent. N-Fbs were found to promote SC proliferation and migration, whereas C-Fbs demonstrated no such effects. This finding suggests that Fbs from different tissue sources can secrete different factors, hence regulating different functions.

The main function of Fbs is to synthesize and secrete extracellular matrix, such as collagen, fibronectin, thrombospondin, and osteopontin, to maintain tissue framework and promote tissue regeneration after injury (Li and Wang, 2011; Lynch and Watt, 2018). Fbs communicate with nearby cells by autocrine, paracrine, and other means of cellular signaling (Sorrell and Caplan, 2009). Researchers suggested some subtypes of Fbs, such as dermal Fbs, could promote nerve regeneration (Y. Hu et al., 2019; Yurie et al., 2021). However, as a well-investigated subtype, C-Fbs have not yet been proven to be correlated with Schwann cells or peripheral nerve regeneration. Therefore, this study was focused on C-Fbs as a subtype for the comparison. To distinguish the factors secreted from N-Fbs versus C-Fbs, GSR-CAA-67 was used to identify 32 DEPs. The top 10 DEPs significantly upregulated in N-Fbs were verified by q-PCR assay, among which GFRa-1, Pdgfa, Ccl11, Lgals3, and Csf2 showed significantly higher expression in N-Fbs compared with C-Fbs. Three DEPs (activin A, eotaxin, and Csf2) reported to be involved in the positive regulation of neuroprotective functions, namely, cell migration, cell proliferation, and neuron differentiation, were selected for ELISA verification. All three DEPs were expressed significantly higher in N-Fbs compared with C-Fbs. It should be noted that Inhba (activin A) was not more highly expressed in N-Fbs according to q-PCR results, but activin A was more highly expressed in N-Fb CM in the ELISA results. We speculate that Inhba expression was largely influenced by post-transcriptional regulation.

Activin A has been reported to promote the migration of various cell types, including colon cancer cells, breast cancer cells, ovarian cancer cells, osteosarcoma cells, and L929 fibroblasts (Zhu et al., 2015; Xie et al., 2017; Yi et al., 2019; Jana et al., 2020; Jiang et al., 2021). Activin A has also been reported to promote the proliferation of prostate cancer cells, cardiac fibroblasts, and leiomyoma cells (Hu et al., 2016; Bao et al., 2018; Chen et al., 2020). This study is the very first to propose that activin A secreted from N-Fbs can facilitate the migration and proliferation of SCs.

SCs play a vital role in the process of injured peripheral nerve repair. After nerve injury, SCs proliferate rapidly and migrate to the injured site, assist macrophages to remove tissue debris and synthesis, and secrete various neurotrophic factors to promote axonal regrowth (Min et al., 2021). In this study, activin A significantly upregulated the proliferation and migration capacity of SCs. Meanwhile, the administration of SB-431542 inhibited such upregulation in SCs. Activin A signals through two type I and two type II receptors (Namwanje and Brown, 2016). Upon ligand binding, activin A activates its kinase activity and phosphorylates the SMAD2/3 intracellular signaling mediators that form a complex with SMAD4, which subsequently translocates to the nucleus and activates or silences gene expression (Massagué and Chen, 2000). Since SB-431542 could abolish the activity of other TGFβ family members, activin A-neutralizing antibodies were used to verify that activin A is a key factor modulating SC proliferation and migration. However, the mechanism of how activin A affects the proliferation and migration of SCs through intracellular signals still needs to be further studied.

In summary, these findings demonstrate that N-Fbs and C-Fbs exhibit different patterns of protein expression. N-Fbs, rather than C-Fbs, can significantly promote the proliferation and migration of SCs. Furthermore, activin A secreted from N-Fbs plays an important role in boosting the proliferation and migration of SCs *in vitro*. *In vivo* results demonstrated an obvious co-localization of activin A and N-Fbs in regenerating sciatic nerves post injury. In future work, we will focus on uncovering more information to clarify the potential mechanisms of activin A and N-Fbs on SC functions and, furthermore, on peripheral nerve regeneration.

## Data Availability Statement

The original contributions presented in this study are included in the article/Supplementary Material, further inquiries can be directed to the corresponding authors.

## Ethics Statement

The animal study was reviewed and approved by Administration Committee of Experimental Animals of Jiangsu Province, China.

## Author Contributions

FD, QH, and YL: study design and manuscript drafting. YL, FY, and ZC: study implementation. YL and QH: data analysis and figure preparation. All authors approved the final manuscript.

## Conflict of Interest

The authors declare that the research was conducted in the absence of any commercial or financial relationships that could be construed as a potential conflict of interest.

## Publisher’s Note

All claims expressed in this article are solely those of the authors and do not necessarily represent those of their affiliated organizations, or those of the publisher, the editors and the reviewers. Any product that may be evaluated in this article, or claim that may be made by its manufacturer, is not guaranteed or endorsed by the publisher.
