# Supplementary material for: Activin A Secreted From Peripheral Nerve Fibroblasts Promotes Proliferation and Migration of Schwann Cells
Source: Front Mol Neurosci. 2022 Jul 7;15:859349. doi: 10.3389/fnmol.2022.859349 (PMC9301483; doi:10.3389/fnmol.2022.859349)
Supplement: Supplementary file 1 [file Table_1.DOCX]

Supplementary Material

**Supplementary Table 1.** List of differentially expressed proteins between N-Fbs and C-Fbs.

| **ProteinID** | **AveExp.N** | **AveExp.C** | **logFC** | **Foldchange** | **Regulation** |
| --- | --- | --- | --- | --- | --- |
| Activin A | 17.84068469 | 14.02540281 | 3.815282 | 14.07713535 | up |
| GFR alpha-1 | 14.08514872 | 11.78793454 | 2.297214 | 4.915077554 | up |
| P-Cadherin | 11.89092826 | 9.784098442 | 2.10683 | 4.307437369 | up |
| PDGF-AA | 15.38071946 | 13.49096508 | 1.889754 | 3.705721311 | up |
| Eotaxin | 14.22813815 | 12.58830963 | 1.639829 | 3.116287888 | up |
| Nope | 12.94539378 | 11.34258922 | 1.602805 | 3.03733189 | up |
| Galectin-3 | 12.78346539 | 11.26822659 | 1.515239 | 2.858461395 | up |
| ICAM-1 | 15.36533117 | 14.10831406 | 1.257017 | 2.390010762 | up |
| Notch-1 | 12.63653163 | 11.4630978 | 1.173434 | 2.255478967 | up |
| GM-CSF | 11.53413654 | 10.41291947 | 1.121217 | 2.175304055 | up |
| TWEAK R | 16.14585718 | 15.11189512 | 1.033962 | 2.047639941 | up |
| IL-1 ra | 12.77676557 | 11.88763172 | 0.889134 | 1.852063861 | up |
| Adiponectin | 10.01884364 | 9.229833089 | 0.789011 | 1.727889011 | up |
| Flt-3L | 13.16575832 | 12.41736137 | 0.748397 | 1.679925147 | up |
| Galectin-1 | 15.00391497 | 14.26722525 | 0.73669 | 1.666347997 | up |
| B7-1 | 11.26369203 | 10.53522076 | 0.728471 | 1.656882465 | up |
| EphA5 | 12.42074362 | 11.79061119 | 0.630132 | 1.547707049 | up |
| IL-17F | 10.05504095 | 9.452817773 | 0.602223 | 1.518054066 | up |
| TIM-1 | 10.62008724 | 10.05921022 | 0.560877 | 1.47516571 | up |
| Neuropilin-1 | 11.13917618 | 10.60470886 | 0.534467 | 1.448407266 | up |
| Notch-2 | 15.80938599 | 15.35728659 | 0.452099 | 1.368029559 | up |
| IL-1a | 11.97122654 | 11.58336424 | 0.387862 | 1.308453178 | up |
| b-NGF | 14.6909567 | 14.36981536 | 0.321141 | 1.249318509 | up |
| Fractalkine | 17.83207342 | 17.565199 | 0.266874 | 1.203198292 | up |
| IL-1b | 11.38476688 | 11.11841394 | 0.266353 | 1.202763454 | up |
| IL-3 | 10.40532281 | 10.6771298 | -0.27181 | 0.828281467 | down |
| TNFa | 14.09443101 | 14.37538582 | -0.28095 | 0.823046124 | down |
| IL-1 R6 | 11.12856893 | 11.50785628 | -0.37929 | 0.768817272 | down |
| Gas 1 | 14.69874461 | 15.22822981 | -0.52949 | 0.692801904 | down |
| MIP-1a | 14.2863297 | 14.87501355 | -0.58868 | 0.664949257 | down |
| JAM-A | 10.33662923 | 10.95563643 | -0.61901 | 0.651118844 | down |
| IL-10 | 13.58259749 | 15.44059777 | -1.858 | 0.275858383 | down |
